# UVA Exposure Combined with Glycation of the Dermis Are Two Catalysts for Skin Aging and Promotes a Favorable Environment to the Appearance of Elastosis

**DOI:** 10.1155/2021/6647773

**Published:** 2021-10-26

**Authors:** Hervé Pageon, Hélène Zucchi, Sylvie Ricois, Philippe Bastien, Daniel Asselineau

**Affiliations:** Department and Institution, L'Oréal Research & Innovation, Avenue Eugène Schueller, Aulnay-sous-Bois 93600, France

## Abstract

Skin aging is the result of superimposed intrinsic (individual) and extrinsic (e.g., UV exposure or nutrition) aging. Previous works have reported a relationship between UV irradiation and glycation in the aging process, leading, for example, to modified radical species production and the appearance of AGEs (advanced glycosylation end products) in increasing quantities, particularly glycoxidation products like pentosidine. In addition, the colocalization of AGEs and elastosis has also been observed. We first investigated the combination of the glycation reaction and UVA effects on a reconstructed skin model to explain their cumulative biological effect. We found that UVA exposure combined with glycation had the ability to intensify the response for specific markers: for example, MMP1 or MMP3 mRNA, proteases involved in extracellular matrix degradation, or proinflammatory cytokine, IL1*α*, protein expression. Moreover, the association of glycation and UVA irradiation is believed to promote an environment that favors the onset of an elastotic-like phenomenon: mRNA coding for elastin, elastase, and tropoelastin expression is increased. Secondly, because the damaging effects of UV radiation *in vivo* might be more detrimental in aged skin than in young skin due to increased accumulation of pentosidine and the exacerbation of alterations related to chronological aging, we studied the biological effect of soluble pentosidine in fibroblasts grown in monolayers. We found that pentosidine induced upregulation of CXCL2, IL8, and MMP12 mRNA expression (inflammatory and elastotic markers, respectively). Tropoelastin protein expression (elastin precursor) was also increased. In conclusion, fibroblasts in monolayers cultured with soluble pentosidine and tridimensional *in vitro* skin constructs exposed to the combination of AGEs and UVA promote an inflammatory state and an alteration of the dermal compartment in relation to an elastosis-like environment.

## 1. Introduction

Chronological or intrinsic aging constitutes the individual and genetic mechanism of aging. A well-known reaction which appears during chronological aging is the glycation reaction. Glycation is a posttranslational modification of proteins, appearing during chronological aging, that results from the reaction between reducing sugar and the amine free function of an amino acid (lysine and arginine) to form AGEs via oxidative or nonoxidative pathways [1]. The presence of these products in the skin changes the physical, biomechanical (stiffening and loss of elasticity), and biological properties (modulation of synthesis and degradation of the matrix by cells) [2]. Another cause of the skin aging process is extrinsic aging, caused by different external factors like UVR (UV radiation and photoaging). This process contributes to the aging mechanism and leads to the elastosis zone in the dermis and also to the formation of wrinkles. Elastosis is observed in the upper reticular zone of the dermis which contains fibers accumulated into amorphous masses mainly composed of elastin [3]. This accumulation is accompanied by degeneration and loss of the surrounding collagen network [4]. It has been reported that the relative amount of elastic tissue in the facial skin increases after the age of 40, and simultaneously, the quantities of type I and III collagen decrease [5]. After UV irradiation, the stimulation of collagen breakdown and inhibition of procollagen synthesis causing loss of collagen content was described to be mediated by matrix metalloproteinase (MMP) expression (e.g., MMP1 and MMP3) [6–8]. Another MMP, MMP12 (human macrophage metalloelastase), which contributes to elastin degeneration, was detected in the superficial dermis in the area of elastotic material and contributes to remodeling in sun-damaged skin [9,10]. These phenomena, chronological and photoaging, can be considered as the two major processes that lead to the global aging of the skin [11, 12]. It has been reported that UV irradiation, particularly UVA irradiation, increases oxidative stress in the dermal matrix and contributes to collagen cross-links [13]. Oxidative stress after UV exposure leads to the generation of reactive oxygen species (ROS) including hydrogen peroxide (H_2_O_2_) [14]. Several damaging effects of ROS have been reported in relation to the photoaging process such as enhanced tropoelastin mRNA levels [15] or induction of MMP1 and MMP3 mRNA and proteins [16, 17]. H_2_O_2_ levels increased after AGEs (e.g., pentosidine) irradiation by UVA and altered the membrane permeability and viability of fibroblasts [18, 19]. In addition, previous studies showed a colocalization between the elastin modifications observed in the photoaging process (actinic elastosis) and AGEs accumulation in this area [20, 21]. Recently, we have shown that photoexposure enhanced AGEs (like pentosidine) preferentially in aged skin, suggesting that a level of AGEs could be required to increase oxidative stress, to potentiate glycoxidation products in photoexposed areas and provoke a negative spiral [22]. The present work reflects the results of several different experiments. First, using our reconstructed skin model modified by collagen glycation [23], the combination of the glycation reaction (chronological and intrinsic aging component) and UVA effects (extrinsic aging component) to mimic the biological effects of these skin aging processes was analyzed. Secondly, because AGEs have been found to be increased in photoexposed skin, the effect of pentosidine on human dermal fibroblasts was investigated.

Our different results lead to the same hypothesis: in the skin, UVA combined with AGEs promotes an environment propitious to the onset of solar elastosis by an inflammatory process and a loss of collagen and elastic network homeostasis.

## 2. Materials and Methods

### 2.1. Skin Samples

Human skin samples biopsied from young (20 yo) and old (75 yo) healthy women were used for the elastin and pentosidine immunostainings. The subjects did not smoke and had Fitzpatrick phototypes II to IV. The skin biopsies (3 mm in diameter) were collected from the ventral and dorsal forearms (considered unexposed and exposed sites, respectively) of each subject. The current revision of the Declaration of Helsinki is accepted as the ethical basis for clinical studies. The study is carried out after approval by an ethics committee (Committee for the Protection of Persons participating in Biomedical Research: CPP) and informed written consent of each participant.

### 2.2. Preglycation of Collagen

Type I bovine collagen (Symatèse, France) was preglycated by incubating it at room temperature with D-ribose 10 mM for one month. At the same time, a collagen control was prepared without D-ribose. After this incubation step, the collagen samples were dialyzed twice against 0.5 N acetic acid solution for 5 days (to remove sugar excess) and three times for 24 hours against 0.017 N acetic acid solution (or 1 : 1000 v/v). Fluorescence was measured at *λ*_ex_ 370 nm/*λ*_em_ 440 nm and *λ*_ex_ 335 nm/*λ*_em_ 385 nm after dialysis to verify the increasing quantity of AGEs in the collagen solution incubated in the presence of ribose.

### 2.3. Cell Culture Isolation and Amplification

Normal human skin was obtained from surgical residues after receiving written informed consent from the donors according to the principles expressed in the Declaration of Helsinki and in article L.1243-4 of the French Public Health Code. Papillary fibroblasts were obtained from breast skin reduction (*n* = 4; age range 19 to 60 yo). The procedure to isolate populations of fibroblasts has been described previously [24]. In brief, using a dermatome, the papillary dermis (superficial layer) was separated from the skin. This dermal section was used to prepare explants that were placed and cultivated in Petri dishes to isolate the corresponding spreading papillary fibroblasts.

Papillary fibroblasts (early passage) were plated onto 150 cm^2^ flasks (Falcon) at the density of 1 × 10^4^ cells/cm^2^ with modified Eagle's medium (MEM, Gibco, Invitrogen) supplemented with nonessential amino acids, L-glutamine, sodium pyruvate (Gibco, Invitrogen), penicillin-streptomycin (20U/ml) (Biochrom, Cambridge Ltd., UK), and 10% fetal calf serum (FCS, Pan Biotech, Dutcher). Cultures were maintained at 37°C in a 95% humidified atmosphere and 5% CO_2_. The medium was renewed three times per week. Fibroblasts were cultivated until subconfluence was reached.

The keratinocytes used in this study were obtained from a single donor (plastic mammary reduction in a 30 yo donor). After extraction from skin [25] and amplification in primary culture [26], the corresponding keratinocytes were frozen in liquid nitrogen until used for 3D skin reconstruction experiments.

### 2.4. Cell Treatment with Pentosidine

Papillary fibroblasts isolated from young (22 yo) or old (60 yo) skin were cultivated in Petri dishes (10,000 cells/cm^2^) with MEM 10% FCS. After 24 h, cells were treated with soluble pentosidine (from PolyPeptide Laboratories, Strasbourg, France) at 1 or 10 *µ*M. The medium was changed every 2 days, and the cells were kept in culture for 1 week.

### 2.5. Reconstructed Skin

Dermal equivalents (collagen-fibroblast contracted lattices) were prepared using human papillary fibroblasts (4 different batches were used independently at 1 × 10^6^ fibroblasts per lattice) embedded into a bovine type I collagen gel (native or collagen modified by glycation). The final volume was 7 ml (with the medium) including 2.1 ml of collagen (concentration 3.5 mg/ml). The lattices were allowed to contract for 5 days at 37°C and 5% CO_2_.

The epidermis was reconstructed by seeding human epidermal keratinocytes on top of the dermal equivalents using stainless rings. The cultures were kept submerged for one week and then raised at the air-liquid interface on grids for one more week to produce a stratified and differentiated epidermis [27]. To cultivate keratinocytes on the lattice and to allow them to form epidermis, the medium used was composed of minimum Eagle's essential medium (MEM) (Gibco, France) supplemented with 10% fetal bovine serum (FBS) (Sigma, France), 10 ng/ml epidermal growth factor (EGF) (Beckton Dickinson, USA), 8.4 ng/ml cholera toxin (Sigma, France), and 0.4 *µ*g/ml hydrocortisone (Sigma, France). The medium was renewed three times per week. Reconstructed skins were irradiated (UVA) with 10 J/cm^2^ using a 1000-Watt xenon arc solar simulator (LOT-Oriel, Palaiseau, France) equipped with a dichroic mirror and a WG-335⁄3 mm filter (Schott, Clichy, France). After irradiation, samples were cultivated in MEM with 1% FCS (to minimize the quantity of endogenous proteins present in the medium). Samples were collected 6 h postirradiation for mRNA analysis or 48 h postirradiation for supernatant analysis and immunostaining of reconstructed skin.

### 2.6. Histology

Samples were fixed in neutral formalin and treated for histology. Paraffin sections (5 *µ*m) were stained with hematoxylin-eosin-saffron (HES) under standard procedures.

### 2.7. Immunohistochemistry

For samples of human skin, double pentosidine/elastin immunolabeling was performed on the 5 *μ*m paraffin sections using mouse monoclonal antibody against pentosidine (KH012, clone PEN-12 from Transgenic, Fukuoka, Japan), developed in VIP (kit Vector SK4600) and rabbit polyclonal antibody against elastin (25011, Novotec, Bron, France), developed in SG (kit Vector SK-4700).

Samples of reconstructed skin were embedded in Tissue-Tek (Miles Inc., Elkhart, IN, USA), frozen in liquid nitrogen, and cut into 5 *µ*m thick sections (cryostat, CM3050 S, Leica, Microsystems, Wetzlar, Germany). Mouse monoclonal antibodies were directed against human fibrillin-1 (1405-01, Southern Biotech, Birmingham, AL, USA) and AGEs (KH001-01, Transgenic, Fukuoka, Japan). Rabbit polyclonal antibodies were directed against human tropoelastin (Protein Resources LLC, Saint Louis, MO, USA). Alexa-conjugated goat antimouse immunoglobulins (Alexa Fluor 488, Invitrogen, Waltham, MA, USA) or fluorescein isothiocyanate (FITC) conjugated swine antirabbit immunoglobulins (F0205, Dako, Glostrup, Denmark) were used as secondary antibodies. Nuclei were stained using propidium iodide (Sigma-Aldrich, Saint Quentin Fallavier, France). Stained tissue sections were observed and imaged under a fluorescence microscope (DMR, Leica, Microsystems, Wetzlar, Germany).

### 2.8. ELISA IL1*α* Assay

The interleukin 1 alpha (IL1*α*) (Quantikine Elisa Kit DLA50, R&D Systems, Minneapolis, MN, USA) content of the tissue culture medium was determined using ELISA assays according to the manufacturer's instructions.

### 2.9. RT-qPCR

Human dermal fibroblasts or reconstructed skin samples were rinsed in Dulbecco's phosphate-buffered saline without calcium and magnesium (Gibco BRL, Cergy Pontoise, France). As for reconstructed skin *in vitro*, the epidermis and dermal equivalents were separated using fine forceps and directly frozen. The different steps for molecular biology analysis (RNA extraction, reverse transcription, and quantitative reverse transcriptase-PCR) were performed by BioAlternatives (Gencay, France). Expression markers were as follows: matrix metalloproteinase 1 (MMP1) (NM_002421), matrix metalloproteinase 3 (MMP3) (NM_002422), macrophage elastase (MMP12) (NM_002426), elastase neutrophil expressed (ELANE) (NM_001972), chemokine (C-X-C motif) ligand 2 (CXCL2) (NM_002089), and interleukin 8 (IL8) (NM_000584) which were analyzed by RT-qPCR from mRNA isolated from the samples. Gene expression was analyzed using the gene PCR array method (specific genes for the dermis or epidermis and 3 housekeeping genes: GAPDH (glyceraldehyde-3-phosphate-dehydrogenase), ACTB (beta-actin), and RPL13 A (ribosomal protein L13 A).

### 2.10. Reverse Transcription

Total cellular RNA was isolated using TRIzol Reagent (Sigma-Aldrich)/chloroform mixture by isopropanol precipitation and extensively treated with DNase I (Kit DNase-free, Ambion). RNA quantity and quality were analyzed using the Bioanalyzer (Agilent Technologies). mRNA was reverse transcribed using the primer oligo(dT) and SuperScript II enzyme (Gibco). cDNA was quantified using the Nanovue (GE Healthcare), and cDNA was adjusted.

### 2.11. Quantitative PCR

cDNAs were subsequently analyzed in duplicate by quantitative real-time PCR using the LightCycler system (Roche Diagnostics, Meylan, France) according to the manufacturer's instructions. For each sample, 2.5 *µ*l of cDNA was mixed with primers and an enzymatic kit (Roche) containing Taq DNA polymerase enzyme, SYBR Green I marker, and MgCl_2_.

Housekeeping mRNAs were quantified in each sample and used for normalization using the REST software.

### 2.12. Statistical Analysis

Results were expressed as the mean ± standard error of the mean or in a box plot representation. An analysis of variance (ANOVA) was performed to assess whether there were differences between the conditions. When significant, ANOVA was followed by adjusted post hoc Tukey–Kramer tests for pairwise comparisons. The two-sided significance level was set at 5%. Data with *p* values <0.05 were considered as significant.

## 3. Results

### 3.1. Specific Fluorescence Measurement Iinduced by AGEs Iincreases in Collagen Samples Modified by Glycation

**Figure 1:**
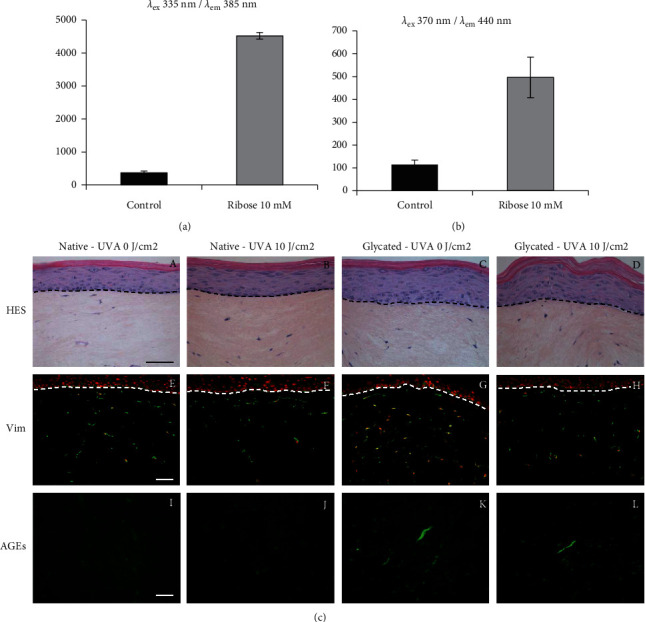
Demonstration of the presence of AGEs and morphology of the reconstructed skin. Fluorescence measurement of collagen solution before reconstruction of the skin at *λ*_ex_ 335 nm/*λ*_em_ 385 nm (a) and *λ*_ex_ 370 nm/*λ*_em_ 440 nm (b). Control: native collagen solution; ribose 10 mM: collagen solution modified by glycation using ribose 10 mM (AU ± SD = arbitrary units ± standard deviation). Reconstructed skin (c): HES histological staining (A to D), vimentin (E to H), and AGEs (I to L) labelling of skin reconstructed *in vitro* using untreated collagen (A, B, E, F, I, J) or preglycated collagen (C, D, G, H, K, L) after (B, F, J, D, H, L) or without (A, E, I, C, G, K) UVA irradiation (10 J/cm^2^) (bar = 100 *µ*m).

The collagen modified by glycation used in glycated *in vitro* skin was prepared before reconstruction as described in Section 2. To estimate the presence of the glycation products in the collagen solution, fluorescence is measured at the end of incubation with the sugar. The fluorescence measurment in the sample containing collagen modified by glycation was higher (×12 and × 4 at *λ*_ex_ 335 nm/*λ*_em_ 385 nm and *λ*_ex_ 370 nm/*λ*_em_ 440 nm, respectively) than in the control sample incubated without ribose (Figures 1(a) and 1(b)). This procedure allows to reveal the presence of AGEs in the collagen modified by glycation.

### 3.2. Histology of Reconstructed Skin Seems Not to Be Aaffected after Low-Dose UVA Irradiation

The morphology of native and glycated reconstructed skin was determined by histological coloration. No change was observed in the morphology between the different native or glycated conditions with or without irradiation (Figure 1(c); A–D). Postexposure (after 48 hours), the viability of fibroblasts seems to be slightly decreased, as shown by the vimentin immunostaining (Figure 1(c); E–H). The presence of AGEs in reconstructed skin was observed by positive immunostaining in the dermal part of the sample containing collagen modified by glycation (Figure 1(c); K, L) when compared to that of the control sample (Figure 1(c); I, J). Overall, this suggests that the morphology and homeostasis of the reconstructed skin does not seem to be altered by these treatments.

### 3.3. AGEs and UVA Irradiation Promote an Environment for Extracellular Matrix Degradation and Inflammation in Reconstructed Skin

**Figure 2:**
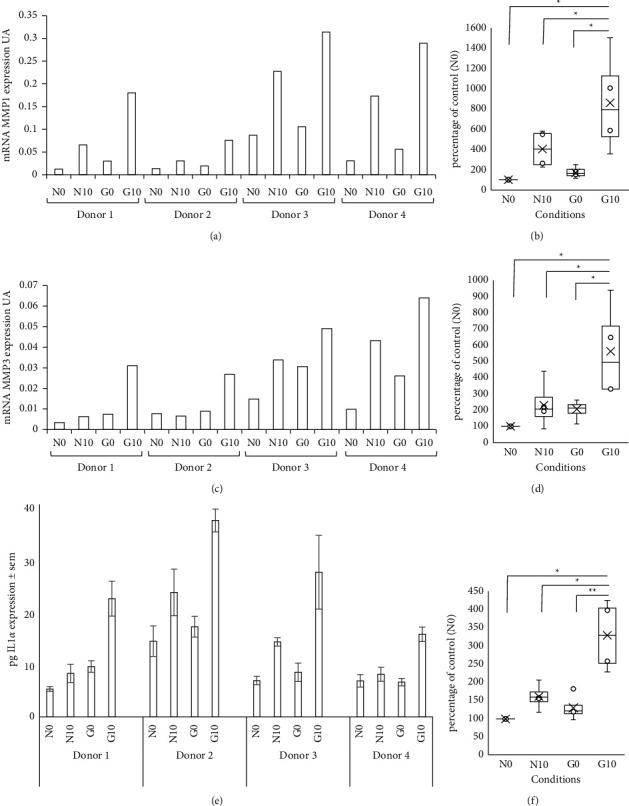
AGEs and UVA irradiation promote an environment for extracellular matrix degradation and inflammation. mRNA MMP1 (a, b) and mRNA MMP3 (c, d) expression measured in dermal layers and interleukin 1 alpha (IL1*α*) detection in the medium (e, f) of reconstructed skin made with fibroblasts isolated from 4 donors separately (a, c, e) or in box plots considering all donors in the same condition group, percentage of control N0 is reported (b, d, f). mRNA levels were quantified from dermal fibroblasts using quantitative reverse transcriptase-polymerase chain reaction (qPCR) 6 hours after UVA irradiation. Each point represents the mean value of normalized mRNA quantity (*n* = 3). Data are expressed in arbitrary units (UA). IL1*α* detection was quantified by the ELISA assay in the medium of reconstructed skin 48 hours after UVA irradiation (in pg IL1*α* per 200 *µ*l of medium) (mean *n* = 3/donors) ± SEM). N0: native collagen without irradiation; N10: native collagen with irradiation (UVA 10 J/cm^2^); G0: glycated collagen without irradiation; and G10: glycated collagen with irradiation (UVA 10 J/cm^2^). Statistics: analysis of variance (ANOVA) adjusted post hoc Tukey–Kramer tests for pairwise comparisons (^∗^*P* < 0.05; ^∗∗^*P* < 0.001).

The aging process is well known to be associated with skin homeostasis modifications and particularly its inflammation and its degradation, explaining why the expression of different markers involved in these processes was given privilege in this study. MMP1 and MMP3 mRNA expression was measured 6 hours after UVA irradiation in fibroblasts from the dermal part of reconstructed skin. In the glycated sample without irradiation, MMP1 and MMP3 mRNA expression was upregulated (×2 and 1.5, respectively, n.s.) compared to the native condition without glycation (N0 vs. G0). UVA irradiation increased MMP1 and MMP3 mRNA expression in the native samples (trend, n.s.) and glycated samples (*p* < 0.05) compared with the nonirradiated conditions (N0 vs. N10 and G0 vs. G10, respectively). If we observed an increase in this expression in the sample containing native collagen in the dermal part after UVA irradiation, this effect was stronger and amplified by the presence of AGEs (*p* < 0.05). Indeed, in glycated samples, MMP1-3 mRNA expression increased by 4.7 (*p* = 0.0036) and 2.7 (*p* = 0.0097) after exposure, respectively (Figures 2(b) and 2(d)), compared to that in the nonirradiated glycated samples (G0 vs. G10). These mRNA protease expression modifications were observed with 4 batches of fibroblasts extracted from 4 different donors (Figures 2(a) and 2(c)).

In the culture medium of reconstructed skin after 48 hours post-UVA exposure, a 1.5-fold increase in IL1*α* secretion (an inflammatory marker) was observed in the culture medium of native *in vitro* skin (ns) induced by irradiation (N0 vs. N10) and strongly amplified by glycation products (G0 vs. G10) (3-fold, *p* = 0.0003) (Figure 2(f)). This effect was observed with the 4 batches of fibroblasts used (Figure 2(e)).

Overall, these results suggested the involvement of AGEs in the promotion of inflammatory and degradation processes in the skin but also in alteration of the ECM with age. These effects were strongly exacerbated when combined with UVA irradiation.

### 3.4. AGEs and UVA Irradiation Promote an Elastosis-Like Environment

**Figure 3:**
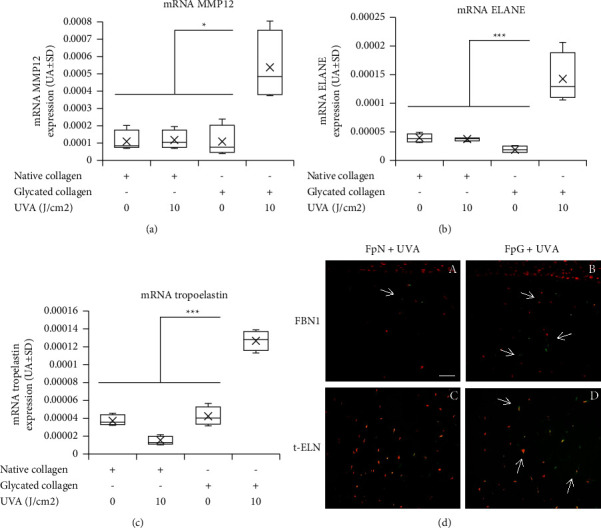
AGEs and UVA irradiation change the elastic network homeostasis and promote an environment in favor of elastosis. mRNA MMP12 (a), elastase (ELANE) (b), and tropoelastin (ELN) (c) expression measured in dermal layers of reconstructed skin made with papillary fibroblasts and native or glycated collagen with or without UVA irradiation (0, 10 J/cm^2^); immunostainings (d) of fibrillin-1 (A, C) and tropoelastin (B, D) of skin reconstructed containing papillary fibroblasts (Fp) (A to D) using native collagen (A, B) or glycated collagen (C, D) after UVA irradiation (10 J/cm^2^). Note that glycation stimulates tropoelastin and fibrillin expression by fibroblasts in 3D culture. mRNA levels were quantified in the dermis (fibroblasts, *n* = 4) using qPCR at the end of the culture emersion phase. Data are expressed in arbitrary units (AU ± SD = arbitrary units ± standard deviation). Statistics: analysis of variance (ANOVA) adjusted post hoc Tukey–Kramer tests for pairwise comparisons (^∗^*P* < 0.05; ^∗∗∗^*P* < 0.0001).

In reconstructed skin, AGEs associated with UVA irradiation lead to upregulation of mRNA expression by MMP12 (+400%, *p* < 0.05) and elastase mRNA (ELANE) (+500%, *p* < 0.0001) 48 hours after exposure (Figures 3(a) and 3(b), respectively). In the same samples, upregulation of tropoelastin mRNA was also observed (+500%, *p* < 0.0001) (Figure 3(c)). This result in reconstructed skin was observed with the combination of three parameters: papillary fibroblasts, AGEs, and UVA. In addition, immunostaining for fibrillin-1 (FBN1) (Figure 3(d); A, B) and tropoelastin (t-ELN) (Figure 3(d); C, D) was only increased in reconstructed skin with the sample containing papillary fibroblasts and glycated collagen after UVA irradiation (Figure 3(d); B, D) compared to that of the control sample (Figure 3(d); A, C). Overall, these results indicated the involvement of AGEs in promoting an elastosis-like environment after exposure to UVA.

### 3.5. AGEs like Pentosidine Stimulate Fibroblasts to Create an Environment That Accentuates Postirradiation Changes

**Figure 4:**
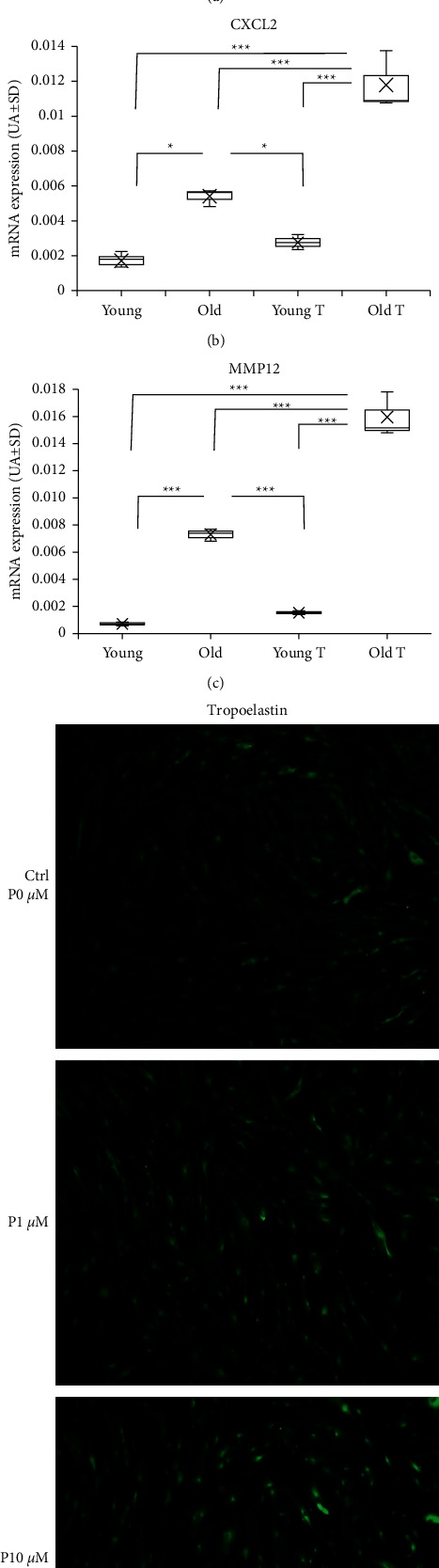
Specific effect of pentosidine on fibroblast monolayers. Determination of IL8 (a), CXCL2 (b), and MMP12 (c) gene expression in fibroblast monolayers (2D culture) from young or old donors after stimulation by pentosidine (young (T) or old (T) during 48 h). mRNA levels were quantified in fibroblasts using qPCR. Each point represents the mean value of normalized mRNA quantity (*n* = 3). qPCR was performed in duplicate on three different samples. Data are expressed in arbitrary units (AU ± SD = arbitrary units ± standard deviation). Tropoelastin immunostaining (d) was observed in fibroblasts monolayers after pentosidine stimulation at 0, 1, and 10 *μ*M. Note that pentosidine stimulates tropoelastin expression by fibroblasts. Statistics: analysis of variance (ANOVA) adjusted post hoc Tukey–Kramer tests for pairwise comparisons (^*∗*^*p* < 0.05; ^*∗∗∗*^*p*< 0.0001).

Indeed, even if we have shown above, with our reconstructed skin model modified by glycation combined with UVA irradiation, that it promotes degradation, inflammation, and a favorable path to the installation of elastosis, the treatment of fibroblasts by pentosidine appears to induce similar changes. In fibroblast monolayer, IL8 mRNA expression was upregulated in fibroblasts from an old donor when compared to a young donor (4.5 fold, *p* = 0.0069). Treatment of both fibroblasts (young and old) with pentosidine induced higher levels of IL8 mRNA (1.5-fold but nonsignificant (n.s.) and 2.9-fold with *p* < 0.0001, respectively) (Figure 4(a)). In addition, CXCL2 mRNA expression increased with age (2.5-fold, *p* = 0.0014) and pentosidine treatment enhanced its expression 1.5- and 2.4-fold for fibroblasts from young (n.s.) and old (*p* < 0.0001) donors, respectively (Figure 4(b)). In aged fibroblasts, MMP12 mRNA expression was upregulated (10-fold, *p* < 0.0001) compared to young fibroblasts (Figure 4(c)). Supplementing the medium with pentosidine enhanced this expression in both fibroblast types by a factor of 2 (*p* < 0.0001) (Figure 4(c)). Tropoelastin protein expression was also enhanced in fibroblast monolayers after treatment with 1 and 10 *µ*M of pentosidine (Figure 4(d)). Overall, these results show that inflammatory and degrading elastin markers are particularly increased with the age of fibroblasts. Treatment with pentosidine exacerbated the expression of these markers and also tropoelastin expression.

## 4. Discussion

Recently, we have demonstrated *ex vivo* that sun exposure promoted the accumulation of pentosidine in the papillary and reticular superior dermis parts of the skin of old donors compared to the nonexposed sites of the same donors and also young donors [22]. Pentosidine accumulation is colocalized with elastin deposition in the elastosis zone, particularly with mature skin. This is in agreement with previous observations that described colocalization between solar elastosis and AGEs [20, 21]. Even if previous observations have shown colocalization between elastosis and glycation, on the other hand, there are few or no studies showing why this process is likely to appear. As a result, the combination of the glycation reaction and UVA effects on the reconstructed skin model to mimic two causes involved in the global aging process (intrinsic and extrinsic aging) and also the impact of pentosidine on fibroblasts in monolayers were investigated. The present study aimed to analyze the biological effects of these skin aging processes together to create a more realistic context and determine whether AGEs could affect the major component of the elastic network. With our reconstructed skin model modified by global glycation (incubation of collagen with sugar to generate several AGE structures), in normal conditions without irradiation, upregulation of MMP protein expression has previously been reported [23]. The literature has reported alterations in gene expression by fibroblasts after irradiation by UVA in reconstructed skin. For example, increasing modulation of MMP1 and MMP3 mRNA expression by fibroblasts in reconstructed skin 6 hours post-UVA exposure has been reported [28,29]. Here, we demonstrated that, although the presence of AGEs or UVA irradiation alone upregulated the expression of the same MMP mRNA, the combination of both emphasized this effect and suggested that extracellular matrix degradation could be more efficient in the photoexposed skin area during extracellular matrix aging. In a similar way, the increasing level of IL1*α* (a proinflammatory cytokine) in the medium of reconstructed skin resulting for both combinations could suggest an important role in the chronic inflammatory process during skin photoaging and could contribute to the inflammaging process [30]. In addition, IL1*α* was reported to stimulate the secretion of CXCL family molecules, playing a role in regulating neutrophil recruitment [31] or in inducing collagenase (MMP1) [17].

Another result emerged from this study, which was the possible involvement of glycation in the extracellular matrix environment in the promotion of the elastosis-like phenomenon. Indeed, elastosis is usually defined as an accumulation of more or less altered elastic material [32, 33] associated with degradation of the collagen network [6, 7]. The elastic material can be modified by glycation [33], and protective molecules like lysozyme which protect elastin from degradation by elastase enzymes can also be modified by glycation (data not shown) and consequently could reduce its protective activity. Indeed, Seité et al. reported that lysozyme deposition on elastotic material was increased [32] and led to resistance from elastase degradation [34]. Here, we showed that the combination of glycation and UVA exposure could induce this process. Glycation and UVA, together with the papillary fibroblast subpopulation in the dermal compartment of reconstructed skin, could promote an environment favoring the onset of an elastosis-like phenomenon. Indeed, mRNA coding for tropoelastin, elastase (ELANE), and MMP12 expression (known to degrade extracellular matrix components such as elastin) was increased compared to the control. Tropoelastin mRNA expression in the fibroblasts of human skin after UV irradiation has been described previously. This may contribute to increased elastin production and contribute to the accumulation of elastotic material [35]. Elastases such as MMP12 were described to increase in photoaged skin and colocalized with solar elastosis material, suggesting a role in the development of this process [36]. Elastin and fibrillin were reported to be substrates for neutrophil elastase (ELANE) [37]. In addition, fibrillin 1 and tropoelastin protein expression were more induced in the conditions containing AGEs and UVA irradiation. Abnormal production of tropoelastin and fibrillin in human skin and their degradation by MMP12 may contribute to the accumulation of elastotic material in photoaged skin [38, 39]. Furthermore, in response to fibrillin fragmentation, upregulation of MMP expression was observed [40] and degradation of decorin by ELANE renders collagen fibrils more susceptible to MMP1 cleavage [41]. The combination of these three parameters (AGEs, UVA, and papillary fibroblasts) seems to be mandatory to promote production and degradation of several elastin network markers at the same time.

In addition, we also demonstrated that pentosidine alone can maintain the fibroblasts in a state of stimulation in favor of alteration of dermal homeostasis. Indeed, some products like pentosidine stimulate fibroblasts to express MMP12 mRNA and tropoelastin protein. It has been previously reported that only some AGEs could provoke tropoelastin synthesis like MGH1 (imidazolone) but not CML (carboxymethyl-lysine) [42]. Therefore, pentosidine accumulation in mature skin, with or without photoexposure, goes on to favor the loss of homeostasis in the elastin network. Pentosidine is a well-known glycoxidation product which is generated by oxidative pathways such as CML, another glycoxidation product. Oxidative stress induced by UVA irradiation [13] could explain in part the increase in pentosidine in the photoexposed site of mature skin [22]. In addition, the present study suggests that pentosidine promotes an environment that favors an inflammatory process. Indeed, after treatment by pentosidine, fibroblasts from young or old donors expressed higher levels of CXCL2 and IL8 mRNA than fibroblasts without treatment. CXCL2 or chemokine (C-X-C motif) ligand 2, also called macrophage inflammatory protein 2-alpha (MIP2-alpha), is involved in many immune responses and is a powerful neutrophil chemoattractant involved in the inflammatory responses [43]. IL8 (as IL6) has been reported to increase with elderly fibroblasts [44] or after UVA exposure in the culture medium of reconstructed skin [45]. This inflammatory effect of pentosidine was previously shown in reconstructed skin with the secretion of MCP1 [46], which is a chemoattractant protein for monocyte lineage cells able to differentiate in dendritic or macrophage cells in contact with AGEs, further aggravating the inflammatory environment [47].

All these results show that these processes are closely interconnected. AGEs (including pentosidine) accumulate in the skin during aging and UVA irradiation increases ROS in the dermis, which promotes the appearance of glycoxidation products such as pentosidine, and consequently contributes to skin aging and elastosis. It is a vicious circle. It is also important to consider the different arrangements of the elastic network within the dermis (mainly composed of elastin and fibrillin). Indeed, the papillary dermis is mostly composed of fibrillin-rich microfibrillar bundles or oxytalan fibers and a finer network of fibers or elaunin fibers, with reduced elastin content running perpendicular to the dermal-epidermal junction (DEJ). While in the reticular dermis, this network is composed of thick elastin-rich fibers running parallel to the DEJ [48]. This distinctive composition of the elastic network in the papillary dermis compared to the reticular dermis means that the latter could facilitate the appearance of the process of elastosis in this area first.

**Figure 5:**
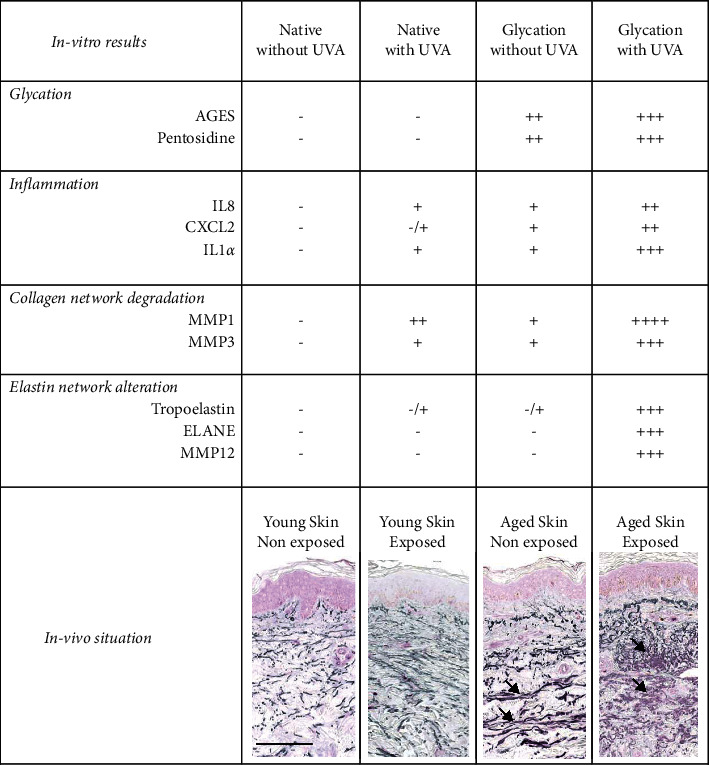
In vitro and in vivo observations related with possible deleterious effect of glycation combined with the UVA irradiation. Elastin (grey) and pentosidine (pink) immunostainings of different forearm human skin. Note that positive labelling for elastin and pentosidine (as shown by the arrow) increased with age and photoexposure (bar = 100 *µ*m). The main changes observed in human skin in function of age and photoexposure that could partly be explained by transposition with our *in vitro* results.

Our observations are summarized in the table (Figure 5), showing the main changes which could be observed after transposition to human skin *in vivo*.

Two points of interest should be considered for future studies. The first would be to investigate the low UVA doses but by a cumulative effect in order to mimic daily irradiation. Indeed, it has been shown that low repeated doses of UVA may lead to photoaging of the skin [49, 50]. The second, because glycation inhibitors are present in food, it would be interesting to investigate these inhibitors or the diet [42] on the severity of the grade of elastosis to reinforce this possible correlation. Indeed, several studies have suggested an effect from diet, associated with lower grades of skin elastosis [51, 52]. The use of sunscreen coupled with the glycation inhibitor or antioxidant effect could prevent the appearance of the elastosis zone. This combination would maintain skin homeostasis for longer and prevent cosmetic problems such as the appearance of wrinkles due to photoexposure.

In conclusion, UVA exposure combined with glycation could amplify the response for specific markers as compared to glycation or UVA exposure alone. Combining glycation and UVA exposure is a useful approach to studying skin aging as shown by increased alteration of the dermal compartment including an elastosis-like phenomenon. Overall, these findings seem to offer a promising way of creating *in vitro* skin models that come so much closer to skin aging *in vivo*, in real life.

## Data Availability

The data used to support the findings of this study are included within the article.

## References

[1] Bailey A. (2001). Molecular mechanisms of ageing in connective tissues. *Mechanism of Ageing and Development*.

[2] Pageon H. (2010). Reaction of glycation and human skin: the effects on the skin and its components, reconstructed skin as a model. *Pathologie Biologie*.

[3] Lavker R. M. (1979). Structural alterations in exposed and unexposed aged skin. *Journal of Investigative Dermatology*.

[4] Schwartz E., Cruickshank F. A., Christensen C. C., Perlish J. S., Lebwohl M. (1993). Collagen alterations in chronically sun-damaged human skin. *Photochemistry and Photobiology*.

[5] El-Domyati M., Attia S., Saleh F. (2002). Intrinsic aging vs. photoaging: a comparative histopathological, immunohistochemical, and ultrastructural study of skin. *Experimental Dermatology*.

[6] Fisher G. J., Choi H.-C., Bata-Csorgo Z. (2001). Ultraviolet irradiation increases matrix metalloproteinase-8 protein in human skin in vivo. *Journal of Investigative Dermatology*.

[7] Marionnet C., Tran C., Bastien P. (2018). A broader filtration of UVA1 wavelengths improves skin photoprotection. *Journal of Dermatological Science*.

[8] Varani J., Spearman D., Perone P. (2001). Inhibition of type I procollagen synthesis by damaged collagen in photoaged skin and by collagenase-degraded collagen in vitro. *American Journal Of Pathology*.

[9] Saarialho-Kere U., Kerkelä E., Jeskanen L. (1999). Accumulation of matrilysin (MMP-7) and macrophage metalloelastase (MMP-12) in actinic damage. *Journal of Investigative Dermatology*.

[10] Tewari A., Grys K., Kollet J., Sarkany R., Young A. R. (2014). Upregulation of MMP12 and its activity by UVA1 in human skin: potential implications for photoaging. *Journal of Investigative Dermatology*.

[11] Zouboulis C. C., Makrantonaki E. (2011). Clinical aspects and molecular diagnostics of skin aging. *Clinics in Dermatology*.

[12] Flament F., Bazin R., Rubert S., Simonpietri V., Piot B., Laquieze B. (2013). Effect of the sun on visible clinical signs of aging in Caucasian skin. *Clinical, Cosmetic and Investigational Dermatology*.

[13] Ou-Yang H., Stamatas G., Kollias N. (2009). Dermal contributions to UVA-induced oxidative stress in skin. *Photodermatology, Photoimmunology & Photomedicine*.

[14] Yaar M., Gilchrest B. A. (2007). Photoageing: mechanism, prevention and therapy. *British Journal of Dermatology*.

[15] Kawaguchi Y., Tanaka H., Okada T. (1997). Effect of reactive oxygen species on the elastin mRNA expression in cultured human dermal fibroblasts. *Free Radical Biology and Medicine*.

[16] Herrmann G., Wlaschek M., Bolsen K., Prenzel K., Goerz G., Scharffetter-Kochanek K. (1996). Photosensitization of uroporphyrin augments the ultraviolet A-induced synthesis of matrix metalloproteinases in human dermal fibroblasts. *Journal of Investigative Dermatology*.

[17] Wlaschek M., Wenk J., Brenneisen P. (1997). Singlet oxygen is an early intermediate in cytokine-dependent ultraviolet-A induction of interstitial collagenase in human dermal fibroblasts in vitro. *FEBS Letters*.

[18] Masaki H., Okano Y., Sakurai H. (1999). Generation of active oxygen species from advanced glycation end-products (AGEs) during ultraviolet light A (UVA) irradiation and a possible mechanism for cell damaging. *Biochimica et Biophysica Acta (BBA) - General Subjects*.

[19] Okano Y., Masaki H., Sakurai H. (2001). Pentosidine in advanced glycation end-products (AGEs) during UVA irradiation generates active oxygen species and impairs human dermal fibroblasts. *Journal of Dermatological Science*.

[20] Mizutari K., Ono T., Ikeda K., Kayashima K.-I., Horiuchi S. (1997). Photo-enhanced modification of human skin elastin in actinic elastosis by N∈-(Carboxymethyl)lysine, one of the glycoxidation products of the maillard reaction. *Journal of Investigative Dermatology*.

[21] Jeanmaire C., Danoux L., Pauly G. (2001). Glycation during human dermal intrinsic and actinic ageing: an in vivo and in vitro model study. *British Journal of Dermatology*.

[22] Bastien P P. H., Poumès-Ballihaut C., Zucchi H., Bastien P., Tancrede E., Asselineau D. (2013). Aged human skin is more susceptible than young skin to accumulate advanced glycoxidation products induced by sun exposure. *Journal of Aging Science*.

[23] Pageon H., Bakala H., Monnier V. M., Asselineau D. (2007). Collagen glycation triggers the formation of aged skin in vitro. *European Journal of Dermatology: EJD*.

[24] Mine S., Fortunel N. O., Pageon H., Asselineau D. (2008). Aging alters functionally human dermal papillary fibroblasts but not reticular fibroblasts: a new view of skin morphogenesis and aging. *PLoS One*.

[25] Didierjean L., Woodley D., Regnier M., Prunieras M., Saurat J. H. (1981). Skin explant cultures: expression of cytoplasmic differentiation antigens in outgrowth cells. *Journal of Investigative Dermatology*.

[26] Rheinwald J. G., Green H. (1975). Serial cultivation of strains of human epidermal keratinocytes: the formation of keratinizing colonies from single cells. *Cell*.

[27] Asselineau D., Bernhard B., Bailly C., Darmon M. (1985). Epidermal morphogenesis and induction of the 67 kD keratin polypeptide by culture of human keratinocytes at the liquid-air interface. *Experimental Cell Research*.

[28] Marionnet C., Lejeune F., Pierrard C., Vioux-Chagnoleau C., Bernerd F. (2012). Biological contribution of UVA wavelengths in non extreme daily UV exposure. *Journal of Dermatological Science*.

[29] Marionnet C., Pierrard C., Golebiewski C., Bernerd F. (2014). Diversity of biological effects induced by longwave UVA rays (UVA1) in reconstructed skin. *PLoS One*.

[30] Goto M. (2008). Inflammaging (inflammation + aging): a driving force for human aging based on an evolutionarily antagonistic pleiotropy theory?. *Biosci Trends*.

[31] Turner N. A., Das A., O’Regan D. J., Ball S. G., Porter K. E. (2011). Human cardiac fibroblasts express ICAM-1, E-selectin and CXC chemokines in response to proinflammatory cytokine stimulation. *The International Journal of Biochemistry & Cell Biology*.

[32] Seite S., Zucchi H., Septier D., Igondjo-Tchen S., Senni K., Godeau G. (2006). Elastin changes during chronological and photo-ageing: the important role of lysozyme. *Journal of the European Academy of Dermatology and Venereology: JEADV*.

[33] Yoshinaga E., Kawada A., Ono K. (2012). N*ε*-(Carboxymethyl)lysine modification of elastin alters its biological properties: implications for the accumulation of abnormal elastic fibers in actinic elastosis. *Journal of Investigative Dermatology*.

[34] Park P. W., Biedermann K., Mecham L., Bissett D. L., Mecham R. P. (1996). Lysozyme binds to elastin and protects elastin from elastase-mediated degradation. *Journal of Investigative Dermatology*.

[35] Seo J. Y., Lee S. H., Youn C. S. (2001). Ultraviolet radiation increases tropoelastin mRNA expression in the epidermis of human skin in vivo. *Journal of Investigative Dermatology*.

[36] Chung J. H., Seo J. Y., Lee M. K. (2002). Ultraviolet modulation of human macrophage metalloelastase in human skin in vivo. *Journal of Investigative Dermatology*.

[37] Kielty C. M., Woolley D. E., Whittaker S. P., Shuttleworth C. A. (1994). Catabolism of intact fibrillin microfibrils by neutrophil elastase, chymotrypsin and trypsin. *FEBS Letters*.

[38] Seité S., Colige A., Deroanne C. (2004). Changes in matrix gene and protein expressions after single or repeated exposure to one minimal erythemal dose of solar-simulated radiation in human skin in vivo. *Photochemistry and Photobiology*.

[39] Chen Z., Seo J. Y., Kim Y. K. (2005). Heat modulation of tropoelastin, fibrillin-1, and matrix metalloproteinase-12 in human skin in vivo. *Journal of Investigative Dermatology*.

[40] Booms P., Pregla R., Ney A. (2005). RGD-containing fibrillin-1 fragments upregulate matrix metalloproteinase expression in cell culture: a potential factor in the pathogenesis of the Marfan syndrome. *Human Genetics*.

[41] Li Y., Xia W., Liu Y., Remmer H. A., Voorhees J., Fisher G. J. (2013). Solar ultraviolet irradiation induces decorin degradation in human skin likely via neutrophil elastase. *PLoS One*.

[42] Pageon H., Zucchi H., Pennacchi P. C., Asselineau D., Farage M. A., Miller K. W., Maibach H. I. (2016). Glycation and skin aging. *In: Text Book of Aging Skin*.

[43] Li J. L., Lim C. H., Tay F. W. (2016). Neutrophils self-regulate immune complex-mediated cutaneous inflammation through CXCL2. *Journal of Investigative Dermatology*.

[44] Wolf J., Weinberger B., Arnold C. R., Maier A. B., Westendorp R. G. J., Grubeck-Loebenstein B. (2012). The effect of chronological age on the inflammatory response of human fibroblasts. *Experimental Gerontology*.

[45] Marionnet C., Grether-Beck S., Seité S. (2011). A broad-spectrum sunscreen prevents UVA radiation-induced gene expression in reconstructed skin in vitro and in human skin in vivo. *Experimental Dermatology*.

[46] Pageon H., Zucchi H., Dai Z. (2015). Biological effects induced by specific advanced glycation end products in the reconstructed skin model of aging. *BioResearch Open Access*.

[47] Pageon H., Zucchi H., Rousset F., Girardeau-Hubert S., Tancrede E., Asselineau D. (2017). Glycation stimulates cutaneous monocyte differentiation in reconstructed skin in vitro. *Mechanism of Ageing and Development*.

[48] Langton A. K., Sherratt M. J., Griffiths C. E. M., Watson R. E. B. (2010). Review Article: a new wrinkle on old skin: the role of elastic fibres in skin ageing. *International Journal of Cosmetic Science*.

[49] Lavker R. M., Gerberick G. F., Veres D., Irwin C. J., Kaidbey K. H. (1995). Cumulative effects from repeated exposures to suberythemal doses of UVB and UVA in human skin. *Journal of the American Academy of Dermatology*.

[50] Lowe N. J., Meyers D. P., Wieder J. M. (1995). Low doses of repetitive ultraviolet A induce morphologic changes in human skin. *Journal of Investigative Dermatology*.

[51] Husein-ElAhmed H., Aneiros-Fernandez J., Gutierrez-Salmeron M. T., Aneiros-Cachaza J., Naranjo-Sintes R. (2013). Relationship between food intake and cutaneous solar elastosis adjacent to basal cell carcinoma. *Journal of the European Academy of Dermatology and Venereology*.

[52] Hughes M. C., Williams G. M., Pageon H., Fourtanier A., Green A. C. (2021). Dietary antioxidant capacity and skin photoaging: a 15-year longitudinal study. *Journal of Investigative Dermatology*.

